# Isoform-Specific Regulation and Localization of the Coxsackie and Adenovirus Receptor in Human Airway Epithelia

**DOI:** 10.1371/journal.pone.0009909

**Published:** 2010-03-26

**Authors:** Katherine J. D. A. Excoffon, Nicholas D. Gansemer, Matthew E. Mobily, Philip H. Karp, Kalpaj R. Parekh, Joseph Zabner

**Affiliations:** 1 Department of Biological Sciences, Wright State University, Dayton, Ohio, United States of America; 2 Department of Internal Medicine, University of Iowa, Iowa City, Iowa, United States of America; 3 Department of Cardiothoracic Surgery, University of Iowa, Iowa City, Iowa, United States of America; Singapore Immunology Network, Singapore

## Abstract

Adenovirus is an important respiratory pathogen. Adenovirus fiber from most serotypes co-opts the Coxsackie-Adenovirus Receptor (CAR) to bind and enter cells. However, CAR is a cell adhesion molecule localized on the basolateral membrane of polarized epithelia. Separation from the lumen of the airways by tight junctions renders airway epithelia resistant to inhaled adenovirus infection. Although a role for CAR in viral spread and egress has been established, the mechanism of initial respiratory infection remains controversial. CAR exists in several protein isoforms including two transmembrane isoforms that differ only at the carboxy-terminus (CAR^Ex7^ and CAR^Ex8^). We found low-level expression of the CAR^Ex8^ isoform in well-differentiated human airway epithelia. Surprisingly, in contrast to CAR^Ex7^, CAR^Ex8^ localizes to the apical membrane of epithelia where it augments adenovirus infection. Interestingly, despite sharing a similar class of PDZ-binding domain with CAR^Ex7^, CAR^Ex8^ differentially interacts with PICK1, PSD-95, and MAGI-1b. MAGI-1b appears to stoichiometrically regulate the degradation of CAR^Ex8^ providing a potential mechanism for the apical localization of CAR^Ex8^ in airway epithelial. In summary, apical localization of CAR^Ex8^ may be responsible for initiation of respiratory adenoviral infections and this localization appears to be regulated by interactions with PDZ-domain containing proteins.

## Introduction

The Coxsackievirus and Adenovirus Receptor (CAR) plays a vital role in cell adhesion and viral infection [1]–[3]. The importance of CAR within epithelial junctions, where it behaves as an adhesion protein interacting with and potentially modulating the trafficking of key PDZ domain containing molecules, is becoming evident [4]–[7]. In contrast, how adenovirus initiates infection of the airway epithelium and whether CAR plays a role in initial adenoviral attachment and infection, when it is sequestered on the basolateral side of airway epithelia, remains unclear [8].

Alternative splicing plays an important role in eukaryotes. During pre-mRNA splicing, the spliceosome cleaves intron sequences, and joins exons together, forming an mRNA. Regulation of the spliceosome can result in alternative splicing of mRNA, determining which exons are present or absent in template mRNA. Alternative splicing not only regulates protein expression, but also allows multiple proteins to be expressed from the same gene resulting in significant proteomic diversity [9]. Alternatively spliced proteins may maintain similar activity, differing only in localization or interactions, or may vary widely in activity or regulation. It is estimated that alternative splicing occurs in 70–80% of human genes, but is more common in regulatory genes, and tissues with diverse cell types [10].

CAR is encoded by a highly conserved, alternatively spliced gene with five described transcripts. Three alternative transcripts encode CAR, lacking the transmembrane domain, yielding a soluble extracellular domain (splicing between exons 4/7, 3/7, 2/7) [11]. In experimental murine models, soluble CAR is able to inhibit viral infection but also results in toxicity [12]–[16]. Although the mechanism of toxicity is unknown, soluble CAR may be predicted to alter CAR-CAR interactions and thus epithelial cell adhesion [17].

Human CAR was first described by Bergelson *et al* as a 7 exon protein [1]. In contrast with other species, mouse CAR (mCAR) was initially cloned as a protein composed of 8 exons [18] and was named mCAR1. The 7 exon mouse form was subsequently identified and termed mCAR2. A detailed analysis of protein expression and localization in mice has revealed differential tissue-dependent expression and localization for the exon 7 and exon 8 isoforms [19], [20]. This suggests that the interactions and potentially the functional importance of these two isoforms may be distinct. Furthermore, considering the emerging importance of signal transduction originating from microdomains within the cell membrane, these two isoforms would be predicted to differentially regulate cellular biology.

The splicing event to create the 8^th^ exon form occurs within the 7^th^ exon. Thus, these two isoforms contain identical extracellular and transmembrane domains, which predicts identical adenovirus binding and serotype preference. The majority of the cytoplasmic domain is identical except for the last 26 (CAR^Ex7^) or 13 (CAR^Ex8^) amino acids. Although comprised of distinct sequences, the last 4 amino acids of both isoforms encode class I PSD95/DlgA/ZO-1 (PDZ) binding domain sequences (-X-(S/T)-X-Ф, where X  =  any amino acid and Ф  =  any hydrophobic amino acid). Interacting PDZ-domain-containing proteins for human CAR^Ex7^ include MAGI-1b, PICK1, PSD-95, MUPP-1, LNX1, and ZO-1 [5]–[7], [21]. Furthermore, murine CAR^Ex7^ and CAR^Ex8^ interact with LNX2 and this interaction appears to be modulated by both the PDZ binding domain of each isoform as well as an upstream sequence common to both isoforms [22].

In contrast with the murine isoform, the protein for human CAR^Ex8^ has never been described. We used a computational approach to identify human exon 8 encoding CAR, and investigated the functional significance of this isoform in primary human airway epithelia.

## Results

### Human CAR exon 8 containing isoform

**Figure 1:**
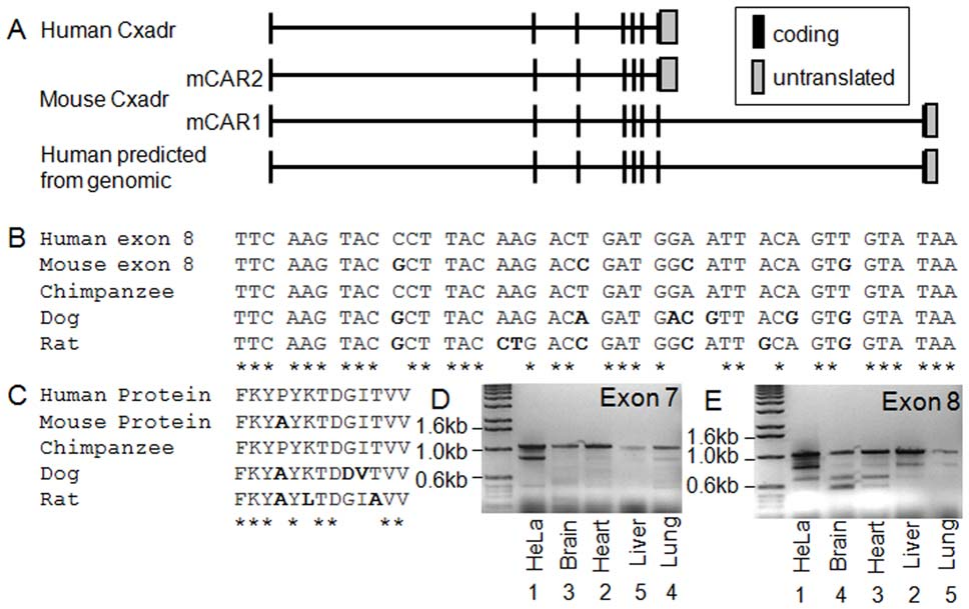
Human CAR is an 8 exon alternatively spliced protein. Panel A shows a schematic diagram of the published and predicted human and mouse exon arrangement. Panel B shows the alignment of the mouse CAR exon 8 with the predicted exon 8 from other species. Panel C and D show representative RT-PCR for human CAR Exon 8 or 7, respectively, in cells (HeLa) or tissues.

Mouse CAR was originally cloned as an 8 exon protein. We hypothesized that human CAR may exist as an 8 exon protein as well (Figure 1A). We first determined whether the human genome contained homologous sequence for exon 8, by performing a BLAT search against the mouse sequence (www.genome.ucsc.edu). Similar sequences were identified for human, chimpanzee, dog and rat (Figure 1B). The predicted amino acid sequence for human and chimpanzee is identical. This sequence differs from mouse by one amino acid. Comparison of hCAR P343 to mCAR A343 using Conseq software identified this amino acid as an exposed or buried residue respectively with a non-structural role. Conservation could not be determined due to insufficient data. The score assigned by Conseq was validated using PolyPhen (http://www.bork.embl-heidelberg.de/polyPhen/), which assigns scores, derived from likelihood matrices, as “benign”, “possibly damaging”, “probably damaging”, or “unknown”. The change P343A was designated as “benign.” Although an intuitive difference between these two amino acids exists, based on these analyses, additional studies to determine whether protein function was affected were not pursued. Primers were designed for RT-PCR to evaluate the presence of hCAR^Ex8^ in several human cell lines including HeLa, 293, A549, Caco-2, and primary human airway epithelia. Transcripts for both hCAR^Ex7^ and hCAR^Ex8^ were detected in all cell lines and primary cells examined (data not shown). A panel of human RNA was subsequently screened for the full-length hCAR^Ex7^ (Figure 1D) or hCAR^Ex8^ (Figure 1E), and yielded full length as well as smaller bands for both transcripts. Semi-quantitative analysis of the bands for hCAR^Ex7^ suggested that heart>brain∼lung>liver. In contrast, hCAR^Ex8^ transcripts showed a rank order of liver∼heart>brain>lung. These data suggest that as in mice, human splice variant expression varies between organs.

### Localization and function of hCAR^Ex8^ is similar to hCAR^Ex7^ in non-polarized cells

**Figure 2:**
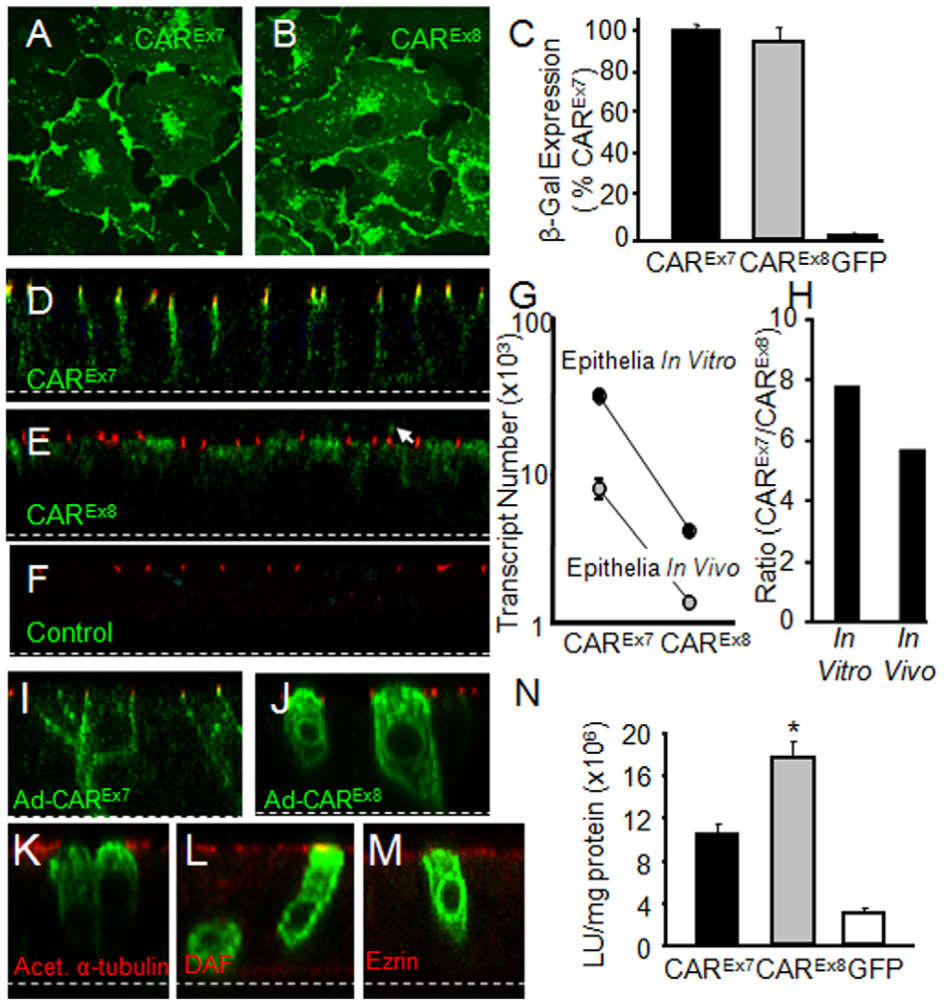
Human CAR^Ex8^ localization and adenovirus-mediated gene transfer is similar to hCAR^Ex7^ in cell monolayers but distinct in polarized cells. COS-7 cells transfected with hCAR^Ex7^ (A) or hCAR^Ex8^ (B) show a similar distribution. Panel C shows that CHO cells transfected with hCAR^Ex7^ or hCAR^Ex8^ mediate similar adenovirus gene transfer. Immunocytochemistry for endogenous hCAR^Ex7^ (D) or hCAR^Ex8^ (E) (green) reveals distinct localization in polarized primary human airway epithelia greater than 2 weeks of age. hCAR^Ex7^ localizes to the basolateral membrane and shows co-localization with the basolateral portion of ZO-1 (red, D, E, F). hCAR^Ex8^ localizes diffusely in the upper region of the cytoplasm with some apical staining (see arrowhead). Panel F shows ZO-1 (red) and a lack of staining with control rabbit pre-immune serum (green). Panel G shows the abundance of CAR^Ex7^ or CAR^Ex8^ transcripts in primary cultures (*in vitro*) or from lung tissue *(in vivo*). Panel H shows that the relative enrichment of CAR^Ex7^ to CAR^Ex8^ transcripts is similar *in vitro* and *in vivo*. Localization of hCAR^Ex7^ (I) or hCAR^Ex8^ (J, K, L, M) after over-expression in primary cultures and co-stained (red) for ZO-1 (I, J), acetylated α-tubulin (K), CD55/decay accelerating factor (DAF, L), or ezrin (M). Panel N shows that expression of exogenous hCAR^Ex8^ in polarized human airway epithelia mediates two-fold greater adenovirus gene transfer than hCAR^Ex7^ in comparison to control GFP transduced cells. *p<0.01. Confocal microscopy (60x oil immersion).

COS-7 cells were transfected with the cDNA for hCAR^Ex7^ (Figure 2A) or hCAR^Ex8^ (Figure 2B). Immuno-localization using the FLAG antibody showed a similar distribution for both with predominant junctional staining in addition to some perinuclear staining. CAR-negative CHO cells transiently expressing hCAR^Ex7^, hCAR^Ex8^, or GFP were infected with recombinant adenovirus containing the bacterial LacZ gene (Figure 2C). As previously described, CHO cells and CHO cells expressing GFP are refractory to adenovirus-mediated gene transfer [23]. In contrast, cells expressing hCAR^Ex7^ or hCAR^Ex8^ showed robust and similar levels of adenovirus-mediated gene transfer proving that both forms of CAR function as adenoviral receptors.

### Localization of hCAR^Ex8^ is distinct from hCAR^Ex7^ in polarized cells

We hypothesized that hCAR^Ex8^ localization may differ from hCAR^Ex7^ in polarized cells. Primary well-differentiated human airway epithelia, greater than two weeks of age, were stained for endogenous hCAR^Ex7^ (Figure 2D, green), hCAR^Ex8^ (Figure 2E, green) with antibodies raised to peptides composed of the last 13 amino acids of either hCAR^Ex7^ or hCAR^Ex8^, or pre-immune serum control (Figure 2F, green), and were co-stained for the tight junction protein ZO-1 (red). Distinct patterns of localization were observed for these two isoforms. As previously shown, hCAR^Ex7^ localizes to the tight and adherens junctions of airway epithelia [23]. In contrast, hCAR^Ex8^ localizes primarily to the upper region of the cytoplasm and apical surface above ZO-1 (Figure 2E, arrow), without ZO-1 tight junction overlap. In contrast to ZO-1, co-localization was observed when co-stained with the apical protein ezrin (Figure S1). To determine the relative abundance of the two isoforms in polarized airway epithelia (*in vitro*) or lung tissue (*in vivo*), RNA was extracted from human airway epithelia greater than two weeks of age or total lung tissue, respectively, and subjected to quantitative RT-PCR (see Figure S2 for primer specificity). Despite donor variability, hCAR^Ex8^ levels were consistently markedly lower than hCAR^Ex7^ both in cultures and tissues (Figure 2G, Figure S2) with a similar ratio of hCAR^Ex7^ to hCAR^Ex8^ both *in vitro* and *in vivo* (Figure 2H). To confirm the distinct localization of these isoforms, airway epithelia, greater than two weeks of age, were transduced from the basolateral side with adenovirus containing the cDNA for hCAR^Ex7^ or hCAR^Ex8^, and subjected to immunocytochemistry 36 hours later. Recombinant expression levels are markedly higher than endogenous levels, thus confocal microscopy settings are set at a level that does not detect endogenous. Nevertheless, the localization of recombinant hCAR was similar to that seen with endogenous isoforms. Whereas the majority of hCAR^Ex7^ localized to the basolateral membrane (Figure 2I), hCAR^Ex8^ was largely diffusely distributed throughout the cell but was also present at the apical membrane where it appeared above ZO-1 (Figure 2J), below the cilia, marked by acetylated α-tubulin (Figure 2K), and overlapped at the same level with apical membrane markers decay accelerating factor (DAF, Figure 2L) and ezrin (Figure 2M) [24]–[26]. We hypothesized that endogenous hCAR^Ex8^ may be responsible for the inefficient, albeit detectable, level of adenovirus infection from the apical surface of airway epithelia. To determine if augmenting expression would augment adenovirus infection, dissociated airway epithelia were transduced with hCAR^Ex7^, hCAR^Ex8^ or GFP and seeded on semi-permeable filters. Cultures were allowed to polarize and form an epithelium over 1 week. When the resistance of all cultures was above 300 mΩ•cm^−2^, cells were infected from the apical surface with adenovirus containing the LacZ gene (Figure 2N). Human airway epithelia expressing GFP showed baseline low level adenovirus-mediated gene transfer. Epithelia expressing hCAR^Ex8^ showed approximately 5-fold greater gene transfer than epithelia expressing GFP (Figure 2N) or mock transduced (Figure S3) and close to a 100% increase in gene transfer compared to epithelia expressing hCAR^Ex7^ (Figure 2N) This increase in infection is similar to previously published results for glycophosphatidylinositol-linked hCAR which is missing the transmembrane and cytoplasmic domains and localizes explicitly to the apical surface of polarized airway epithelia [27]. This suggests that hCAR^Ex8^ explains the low-level baseline apical adenovirus infection and that there may be a maximal amount of infection possible through apically localized receptor. These data also raise the question why hCAR^Ex8^ does not localize to the basolateral surface.

### hCAR^Ex8^ interacts with PSD-95 but not PICK1 via the PDZ binding domain

**Figure 3:**
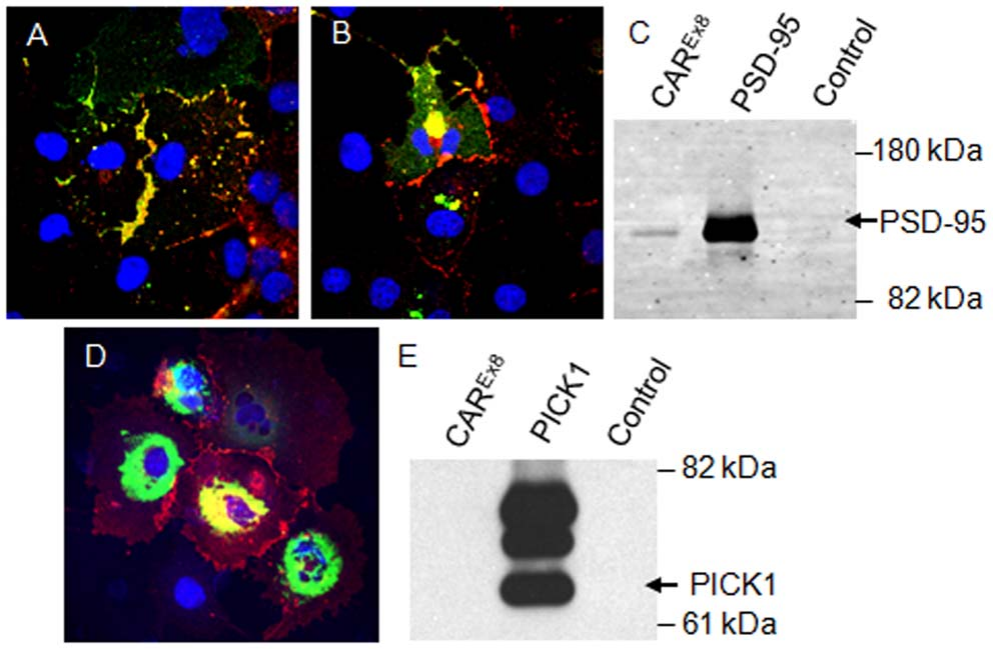
hCAR^Ex8^ co-localizes and interacts with PSD-95 but not PICK1. Panel A shows co-localization (yellow) of hCAR^Ex8^ (red) and PSD-95-GFP (green). In contrast, in panel B, hCAR^Ex8-PDZ^ does not co-localize at the junctions of cells. hCAR^Ex8-PDZ^ localizes to the junctions between cells whereas PSD-95-GFP fluorescence remains diffuse. Panel C shows immunoprecipitation of PSD-95-GFP with the hCAR specific extracellular domain monoclonal antibody RmcB, GFP antibody, but not a control antibody (MopC). Panels D and E shows the lack of co-localization and immunoprecipitation between hCAR^Ex8^ (junctional) and PICK1-GFP (perinuclear). Confocal microscopy (60x oil immersion).

PDZ-interactions may modulate localization as well as function. It is known that both the sequence of the binding domain and the upstream sequences affect PDZ domain interactions. The mechanism by which the upstream sequences affect the specificity of interaction remains unclear. Although hCAR^Ex7^ and hCAR^Ex8^ have type 1 PDZ binding domains (X(S/T)XΦ) at the C-terminus (hCAR^Ex7^ –GSIV; hCAR^Ex8^ –ITVV), these and the upstream sequences are distinct. Thus we hypothesized that PDZ-interactions may be responsible for altered localization in human airway epithelia. We have previously shown that hCAR^Ex7^ interacts with PICK1 and PSD-95 via a PDZ binding domain specific interaction such that co-localization by immunocytochemistry reveals hCAR^Ex7^ is able to pull these proteins out of the cytoplasm and co-localize at the junctions between cells. We further hypothesized that hCAR^Ex8^ may interact with some hCAR^Ex7^ partners. COS-7 cells were co-transfected with hCAR^Ex8^ and PSD-95-GFP and immunocytochemistry was performed to determine localization. hCAR^Ex8^ localized to the junctions as observed in Figure 2B. In the absence of hCAR^Ex8^, PSD-95-GFP localizes diffusely within the cytoplasm [6]. In the presence of hCAR^Ex8^, the localization of PSD-95-GFP was altered to co-localize with hCAR^Ex8^ at the junctions of cells (Figure 3A). Localization of a PDZ mutant form of hCAR^Ex8^ (hCAR^Ex8-PDZ^) was similar to full length hCAR^Ex8^ (Figure 3B) and was unable to alter the diffuse localization of PSD-95-GFP upon co-transfection. Whereas an interaction with full length hCAR^Ex8^ was evident by co-immunoprecipitation (Figure 3C), no interaction was observed by coimmunoprecipitation with hCAR^Ex8-PDZ^, indicating that this interaction requires the ITVV PDZ binding domain sequence. Next, hCAR^Ex8^ and PICK1-GFP were co-transfected into COS-7 cells. Immunocytochemistry for hCAR^Ex8^ revealed the lack of interaction between PICK1-GFP and CAR^Ex8^ at the junctions where the majority of hCAR^Ex8^ was localized (Figure 3D). In some cells there was co-localization in the perinuclear region. To determine if there was a physical interaction, each protein was immunoprecipitated and evaluated by Western blot (Figure 3E). No interaction was detected. Taken together, these data suggest that either there is no interaction or the interaction is too weak to pull PICK1-GFP to the intercellular junctions. Co-localization of hCAR^Ex8^ and PICK1-GFP in the perinuclear region may represent an artifact of high protein expression or alternatively PICK1-related retention of CAR^Ex8^.

### hCAR^Ex8^ interaction with MAGI-1b-GFP results in hCAR^Ex8^ degradation

**Figure 4:**
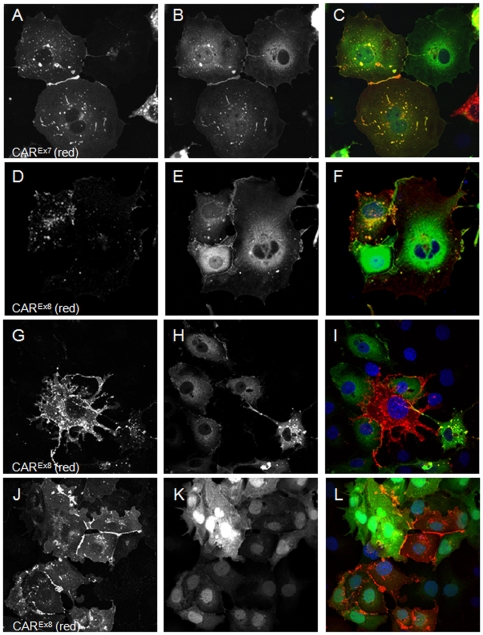
Co-expression of hCAR^Ex8^ and MAGI-1b-GFP results in the loss of hCAR^Ex8^. In contrast to the co-localization of hCAR^Ex7^ (A, red) and MAGI-1b-GFP (B, green) as shown in panel C (yellow), co-expression of hCAR^Ex8^ (D, G, red) and MAGI-1b-GFP (E, H, green) results in decreased levels of hCAR^Ex8^ (F) unless MAGI-1b-GFP is absent from the cell (I). Co-expression of hCAR^Ex8^ (J, red) with GFP (K, green) results in abundant hCAR^Ex8^ expression at the junctions of the cells and diffuse GFP expression (L). Confocal microscopy (60x oil immersion).

hCAR^Ex8^ differentially interacts with hCAR^Ex7^ PDZ-mediated interacting partners despite having a similar class of PDZ binding domain. We have previously shown an interaction between hCAR^Ex7^ and MAGI-1b-GFP (Figure 4A-C). To investigate the interaction between hCAR^Ex8^ and MAGI-1b-GFP, COS-7 cells were co-transfected and evaluated by immunocytochemistry. Surprisingly, little to no hCAR^Ex8^ staining (Figure 4D) was present in cells expressing MAGI-1b-GFP (Figure 4E). Most of the small amount of hCAR^Ex8^ present within MAGI-1b-GFP positive cells appeared within vesicular structures and not the junctions (Figure 4F). The presence, localization and amount of hCAR^Ex8^ appeared to be dependent on the amount of MAGI-1b-GFP. A small percentage of cells appeared to express hCAR^Ex8^ alone and the expression was robust in comparison to neighboring MAGI-1b-GFP expressing cells (Figure 4G-I).This was not the case for PSD-95-GFP, PICK1-GFP, or GFP (Figure 4G-I). hCAR^Ex8^ was detectable by Western blot, presumably due to the relatively few cells transfected with hCAR^Ex8^ but not MAGI-1b-GFP. No interaction between hCAR^Ex8^ and MAGI-1b-GFP was observed by co-immunoprecipitation even when cells were treated with proteosomal inhibitors (data not shown). These data suggest that a transient interaction between MAGI-1b and hCAR^Ex8^ results in the disappearance of hCAR^Ex8^.

### Quantitation of the stoichiometric hCAR^Ex8^ interaction with MAGI-1b-GFP

**Figure 5:**
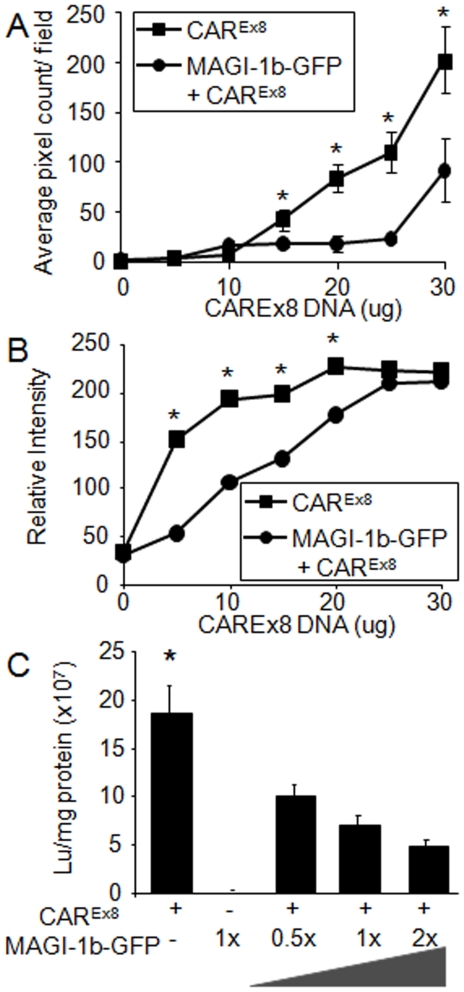
Co-expression of hCAR^Ex8^ with MAGI-1b-GFP results in less immunofluorescence, protein, and adenovirus-mediated gene transfer. COS-7 cells were transfected with 0 to 30 µg of hCAR^Ex8^ +/− 15 µg MAGI-1b-GFP and evaluated for hCAR^Ex8^ specific immunofluorescence (A) or CAR^Ex8^ specific protein by Western blot (B). In the presence of MAGI-1b-GFP, the hCAR^Ex8^ expression curve is shifted to the right suggesting a loss of hCAR^Ex8^ protein. Panel C shows CHO cells transfected with varying amounts of hCAR^Ex8^ and MAGI-1b-GFP, and evaluated for Ad-β-galactosidase gene transfer. Co-expression of MAGI-1b-GFP resulted in a decrease of adenovirus-mediated gene transfer in a dose response relationship. *p<0.03.

Since some MAGI-1b-GFP expressing cells also expressed some, albeit mislocalized, hCAR^Ex8^, we hypothesized that there would be a stoichiometric relationship between these proteins. COS-7 cells were electroporated with a dose response of hCAR^Ex8^ (0-30 µg plasmid DNA) in the presence of constant (15 µg plasmid) MAGI-1b-GFP or GFP. hCAR^Ex8^ expression was evaluated by immunocytochemistry (red fluorescence) using fluorescence microscopy and Western blot. Fluorescence intensity was calculated as average pixel count per field of view (n = 6, Figure 5A). Increasing the amount of hCAR^Ex8^ plasmid, in the presence of constant GFP plasmid, resulted in a relatively linear increase in hCAR fluorescence (Figure 5A). In contrast, when hCAR^Ex8^ was co-transfected with a constant amount of MAGI-1b-GFP, the dose response curve was shifted significantly to the right, indicating that hCAR^Ex8^ expression was suppressed in the presence of MAGI-1b-GFP relative to co-expression with GFP. Western blot using the hCAR^Ex8^ specific antibody was quantitated by chemiluminescence imaging, relative to β-actin protein expression (Figure 5B), and revealed a similar relationship. Although a rapid increase in hCAR^Ex8^ protein in the presence of GFP was observed, the sensitivity of detection was apparent by the plateau of the curve. The dose response curve for hCAR^Ex8^, when co-transfected with MAGI-1b-GFP, was again shifted significantly to the right. The observed difference in hCAR^Ex8^ protein was not due to transfection or transcription since quantitative PCR for plasmid and RT-PCR for hCAR^Ex8^ RNA were similar (data not shown). Moreover, no effect was observed on GFP or MAGI-1b-GFP fluorescence upon increasing hCAR^Ex8^ (data not shown).

### MAGI-1b-GFP interaction with CAR^Ex8^ decreases adenovirus infection

To determine a physiological response to the loss of hCAR^Ex8^ in the presence of MAGI-1b-GFP, CHO-K1 cells were transfected with hCAR^Ex8^ or MAGI-1b-GFP, or co-transfected with hCAR^Ex8^, at a constant dose, with MAGI-1b-GFP at increasing doses. All transfections were balanced with GFP plasmid to maintain equal amounts of DNA. Transfected cells were transduced with Ad-β-gal 48h later. β-galactosidase expression was determined 24 hr post-transduction (Figure 5C). Similar to Figure 2C, transfection of CHO-K1 cells with hCAR^Ex8^ renders them susceptible to adenovirus infection while transfection with MAGI-1b-GFP does not. Co-transfection of hCAR^Ex8^ with a dose response of MAGI-1b-GFP resulted in a dose-related reduction of susceptibility to Ad-βgal-mediated gene expression, indicating that there was a reduction of cell surface hCAR^Ex8^ available as a receptor. Taken together with the previous data, we concluded that in contrast to hCAR^Ex7^, co-expression of hCAR^Ex8^ with MAGI-1b-GFP results in the disappearance of hCAR^Ex8^ and may explain the absence of hCAR^Ex8^ in the adherens junctions of airway epithelia.

### The hCAR^Ex8^-MAGI-1b-GFP interaction requires the hCAR^Ex8^ PDZ binding domain

**Figure 6:**
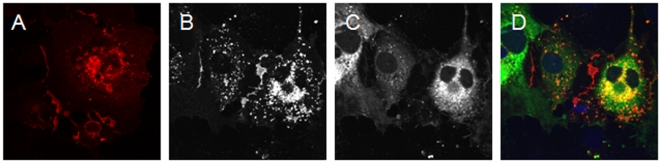
MAGI-1b-GFP-mediated loss of hCAR^Ex8^ requires the PDZ-binding domain (ITVV) of hCAR^Ex8^. COS-7 cells were transfected with hCAR^Ex8-PDZ^ (A, red) or co-transfected with hCAR^Ex8-PDZ^ (B, red) and MAGI-1b-GFP (C, green). Panel D shows the lack of co-localization of hCAR^Ex8-PDZ^ (junctions) and MAGI-1b-GFP (cytoplasmic/diffuse). Confocal microscopy (60x oil immersion).

The interaction between the hCAR^Ex7^ isoform with MAGI-1b-GFP requires the CAR^Ex7^ PDZ binding domain (-GSIV). To determine the requirement of the hCAR^Ex8^ PDZ binding domain, a stop codon was added to the cDNA of hCAR^Ex8^ by site-directed mutagenesis resulting in a protein missing the last 4 amino acids (-ITVV). Similar to wild-type hCAR^Ex8^ (Figure 2B), COS-7 cells transfected with hCAR^Ex8-PDZ^ showed robust junctional localization with some protein within intracellular vesicles (Figure 6A). Co-transfection of hCAR^Ex8-PDZ^ with MAGI-1b-GFP resulted in co-expression of both proteins and did not alter the junctional localization of hCAR^Ex8-PDZ^ (Figure 6B) or show diffuse localization of MAGI-1b-GFP (Figure 6C). These data indicate that the PDZ binding domain is required for the disappearance of hCAR^Ex8^. Thus, an interaction between hCAR^Ex8^ and MAGI-1b is required, raising the question of how this interaction differs from hCAR^Ex7^ and MAGI-1b.

### The upstream sequence plays a role in PDZ interactions

**Figure 7:**
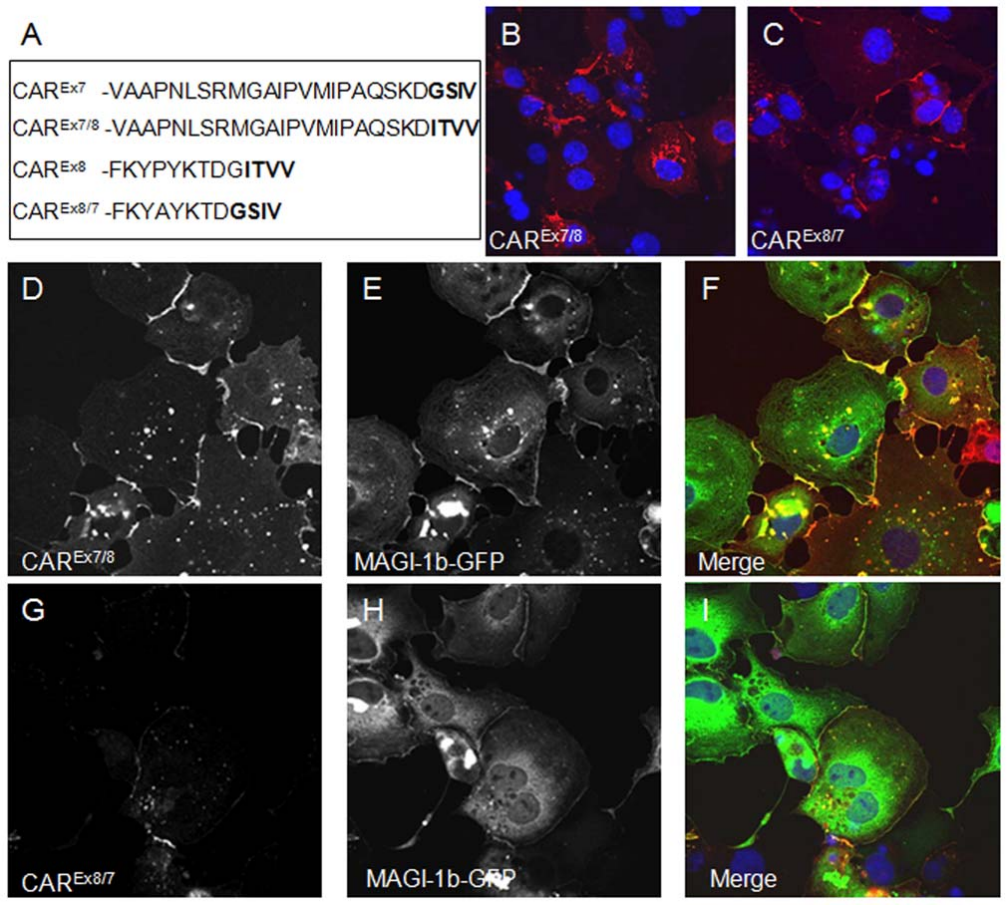
The result of the MAGI-1b-GFP interaction with hCAR requires both the PDZ binding domain and the upstream isoform specific amino acids. The PDZ binding domain of hCAR^Ex7^ and hCAR^Ex8^ were swapped by PCR as shown in panel A. Both constructs contained identical upstream sequences. The localization was determined in transfected COS-7 cells either alone, hCAR^Ex7/8^ (B), hCAR^Ex8/7^ (C) or upon co-transfection with MAGI-1b-GFP (D-I). Panels D and E show junctional expression of hCAR^Ex7/8^ and MAGI-1b-GFP, respectively, and co-localization in panel F. Panel G shows minor expression of hCAR^Ex8/7^ in the presence of robust MAGI-1b-GFP expression in panel H. Some co-localization is observed in panel I. Confocal microscopy (60x oil immersion).

**Figure 8:**
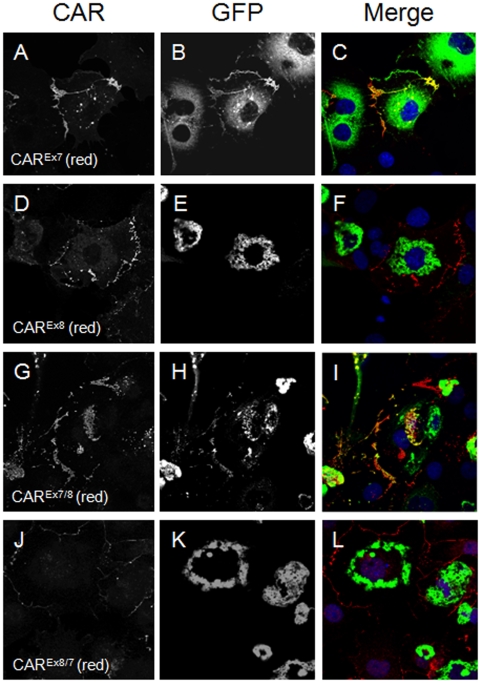
The interaction between the hCAR PDZ binding domain and PICK1 depends on both the PDZ binding domain and the upstream isoform specific amino acids. The PDZ binding domain of hCAR^Ex7^ and hCAR^Ex8^ were swapped by PCR as shown in Figure 7, panel A. The localization was determined in transfected COS-7 cells. hCAR^Ex7^ (A) transfected with PICK1-GFP (B) results in co-localization at the junctions (C, yellow). hCAR^Ex8^ (D) transfected with PICK1-GFP (E) results in no co-localization at the junctions (F). hCAR^Ex7/8^ (G) transfected with PICK1-GFP (H) results some co-localization at the junctions (I, yellow). hCAR^Ex8/7^ (J) transfected with PICK1-GFP (K) results in no co-localization at the junctions (L). Confocal microscopy (60x oil immersion).

We have previously shown a PDZ dependent interaction between hCAR^Ex7^ and PICK1-GFP [6]. Figure 3D demonstrates that there is no co-localization, and Figure 3E, shows there is no co-immunoprecipitation when hCAR^Ex8^ and PICK1-GFP are co-expressed. hCAR^Ex7^ and hCAR^Ex8^ differ by only the terminal 26 or 13 amino acids respectively. Since both hCAR^Ex7^ and hCAR^Ex8^ have type I PDZ binding domains we asked whether the PICK1 interaction was dependent on the terminal 4 amino acid sequence or the unique upstream sequences. To further define the role these sequences play in the PDZ domain-PDZ binding domain interaction, the hCAR^Ex7^ PDZ binding domain was swapped with the hCAR^Ex8^ PDZ binding domain (i.e. 22aa of hCAR^Ex7^ followed by ITVV, hCAR^Ex7/8^) or the hCAR^Ex8^ was swapped with hCAR^Ex7^ (i.e. 9aa of hCAR^Ex8^ followed by GSIV, hCAR^Ex8/7^). hCAR^Ex7^, hCAR^Ex8^, hCAR^Ex7/8^, or hCAR^Ex8/7^ were each co-transfected with PICK1-GFP. As previously shown, co-transfection of COS-7 cells with hCAR^Ex7^ and PICK1-GFP results in accumulation of hCAR^Ex7^ at the junctions of cells (Figure 7A) and hCAR^Ex7^ is able to pull PICK1-GFP (Figure 7B) from a perinuclear localization to the junctions of cells (Figure 7C). In contrast, as also demonstrated in Figure 3D, co-transfection of COS-7 cells with hCAR^Ex8^ and PICK1-GFP results in accumulation of hCAR^Ex8^ at the junctions of cells (Figure 7D). However, PICK1-GFP (Figure 7E) remains in a perinuclear localization (Figure 7F). Transfection of either hCAR^Ex7/8^ (Figure 7G) or hCAR^Ex8/7^ (Figure 7H) alone resulted in junctional localization. Interestingly, co-transfection of COS-7 cells with hCAR^Ex7/8^ and PICK1-GFP results in the co-localization of hCAR^Ex7/8^ (Figure 7I) and PICK1-GFP (Figure 7J) at the junctions of cells (Figure 7K). This suggests that the sequence ITVV is able to interact with PICK1-GFP but not in the context of the upstream hCAR^Ex8^ unique sequence. Finally, co-transfection of COS-7 cells with hCAR^Ex8/7^ and PICK1-GFP results in accumulation of hCAR^Ex8/7^ at the junctions of cells (Figure 8L), while PICK1-GFP (Figure 8M) remains in a perinuclear localization in a manner similar to wild type hCAR^Ex8^ (Figure 8N). These data indicate that the upstream sequence plays a significant role in the specificity of the PDZ domain-PDZ binding domain interaction and thus interactions must be defined experimentally.

### hCAR^Ex8^ PDZ binding domain and upstream nine amino acids define the MAGI-1 interaction

The above data indicates that sequence immediately upstream of the PDZ binding domain may define the PDZ interaction. The difference between the respective interactions of hCAR^Ex7^ or hCAR^Ex8^ with MAGI-1b-GFP may also be due to interactions with the upstream 22 or 9 amino acids, the 4 terminal PDZ binding domain amino acids or a combination of both. Co-transfection of hCAR^Ex7/8^ with MAGI-1b-GFP resulted in the relocation and co-localization of MAGI-1b-GFP with hCAR^Ex7/8^ at the junctions of cells (Figure 8A-C). Interestingly, co-transfection of hCAR^Ex8/7^ with MAGI-1b-GFP revealed an intermediate phenotype (Figure 8D-F). Although there were depressed levels of hCAR^Ex8/7^ within the population of transfected cells (Figure 8D), some cells showed low levels of hCAR^Ex8/7^ expression at the junctions and low levels of co-localization with MAGI-1b-GFP at these junctions. Taken together with the results in Figure 7, these data suggest that although the PDZ binding domain is required for the interaction, both the exact sequence of the binding domain and upstream sequences may modulate the interactions of PDZ domain containing proteins.

## Discussion

Adenoviruses are among the most studied viruses from the viral biology, cell biology and pathogenesis perspective. However, the failure of these viruses in applications for airway gene therapy and the identification of a basolaterally localized receptor, hCAR, made it clear that apical infection is inefficient. We and others found that once an epithelial cell is infected, progeny viruses can easily infect neighboring cells via the basolateral route, and moreover, disruption of cell adhesion by fiber allows virus escape into the lumen of the airway and back into the environment [4]. The question remained: is the initial infection random, requiring damage or does it use the adenovirus fiber-knob or another receptor?

Human transcripts containing the predicted exon 8 have been described [2], [28] but protein presence never verified. To better understand the function of hCAR we sought to investigate the presence, localization and interactions of human CAR^Ex8^ and their relevance in the human airway epithelium.

Proteins exhibit regulation at multiple levels. Here we show that not only is the transmembrane form of human CAR regulated by splicing, but also the exon 8 specific isoform is subsequently regulated at the protein level by the PDZ domain containing protein MAGI-1b. Furthermore, hCAR^Ex8^ localizes to the apical compartment of human airway epithelia where it could serve as the receptor initiating adenovirus infection.

RNA splicing is an important event that significantly increases proteomic diversity and may impart cell specificity. Despite the importance of hCAR in viral infection, cell adhesion, and development, the genetic and splicing regulation of the gene for hCAR, *CXADR*, have not been well studied. It is known that expression of hCAR is upregulated by histone deacetylase (HDAC) inhibitors, a finding with significant implications for oncolytic adenoviral cancer therapy [29]–[31]. Whether HDAC inhibitors affect the splicing of hCAR is also currently unknown.

Several transcripts have been described for hCAR. However, the importance of each splice variant is unclear. Secreted splice variants that interact with the extracellular domain of the transmembrane form of hCAR could clearly alter homophilic transmembrane hCAR interactions and hence modulate junctional remodeling. These variants could also play a physiological role modulating interactions between hCAR and its ligand, AMICA1/JAML, found on transmigrating lymphocytes and dendritic cells [32].

Considering the importance of junctional organization, interactions, and signaling, alternative transmembrane forms could play equally important roles in junctional remodeling and responses to ligation with soluble isoforms or ligands on other cell types. Two transmembrane isoforms are known for CAR. Although several interactions have been discovered for both the mouse and human CAR^Ex7^ isoform, only one interaction has been described for the alternative, less prevalent mouse CAR^Ex8^ isoform. Both mouse CAR^Ex7^ and CAR^Ex8^ interact with Ligand-of-Numb Protein-X2 (LNX2), an intracellular scaffolding protein that may play a role in Notch signaling [22]. Interestingly, both isoforms interact with LNX2 through two different regions within the intracellular domain of mCAR; each unique C-terminal PDZ binding domain and a region in common just upstream of the splice junction.

Our previous work revealed that interaction with hCAR^Ex7^ results in junctional localization of MAGI-1b [6]. In contrast, this work shows that the interaction with hCAR^Ex8^ is unique to other MAGI-1b interactions since it results in the loss of hCAR^Ex8^. Epithelia express MAGI-1b where it localizes to the basolateral junctions. hCAR^Ex7^ also localizes to the basolateral junctional adhesion complex. The data presented herein is consistent with MAGI-1b interacting with hCAR^Ex7^ at the junctions between epithelial cells. It is also consistent with the unexpected apical localization of hCAR^Ex8^. If hCAR^Ex8^ were to go to the basolateral junctions, it would be predicted to interact with MAGI-1b and be degraded. Thus the only place it could exist in the cell is in an apical compartment devoid of MAGI-1b. Alternatively, there may be a specific mechanism for the apical localization of hCAR^Ex8^ that remains to be discovered. Apical localization is consistent with elevated adenovirus infection after expression of hCAR^Ex8^. Thus this isoform of hCAR would be able to interact with adenovirus on the luminal air-exposed surface and mediate the initial infection of an epithelium. It is notable that the increase of infection is similar to the extracellular domain of hCAR conjugated to a glycophosphatidyl-inositol (GPI) tail, which explicitly is apically localized in airway epithelia [27], [33]. *In vitro*, the C-terminus of hCAR is not required for adenovirus infection [27], however, considering the distinct localization and interactions between these isoforms, we cannot predict whether infection is identical *in vivo*. Furthermore, it is interesting to speculate that mutations altering the splicing or transcript abundance of the hCAR^Ex8^ isoform may be responsible for viral susceptibility.

PDZ-based regulation has been described for other membrane proteins [34], [35]. For example several PDZ-domain containing proteins are known to interact with the cystic fibrosis transmembrane conductance regulator (CFTR) [36], [37]. In contrast to hCAR^Ex7^, CFTR traffics to the apical membrane of airway epithelia where it behaves as a chloride channel. Interactions between the PDZ-binding domain of CFTR and the PDZ domain of Na^+^/H^+^ exchanger-3 regulatory factors 1 and 2 (NHERF1 and NHERF2) act to stabilize CFTR at the cell surface [38]. CFTR PDZ interactions with the CFTR-associated ligand (CAL) target CFTR for lysosomal degradation [39]. Cushing *et al* have recently shown that the delicate balance of interactions regulating cell surface maintenance and lysosomal degradation upon cycling is due to the relative affinity of the PDZ interactions [37]. The stronger interaction between CFTR and NHERF1/2 would be predicted to out compete the weaker interaction with CAL resulting in the relatively long CFTR half-life observed. This type of regulation differs from our data in at least two ways. One isoform (CAR^Ex7^) appears to be dominant in directing the localization of the PDZ-domain containing proteins while MAGI-1b is dominant in the interaction with hCAR^Ex8^ resulting in the loss of this protein. To our knowledge, all descriptions of PDZ-directed degradation have been during mature protein cycling. In contrast, we have observed immediate loss of hCAR^Ex8^ implying regulation during early protein synthesis or quality control stages.

The question remains why differential compartmentalization of these hCAR isoforms would exist. The signals transduced by these isoforms, despite having the same extracellular ligands, may differ. Differential localization of these two isoforms could result in the cell being able to discern whether the signal is from the apical or basolateral compartment and mount the appropriate response. For example, both isoforms would recognize AMICA1/JAML present on the surface of neutrophils and dendritic cells. hCAR^Ex7^ may be a gatekeeper for neutrophil transmigration but hCAR^Ex8^ may either tether neutrophils to the apical surface or sense how many neutrophils are present. Alternatively, these two isoforms may play a role in dendritic cell surveillance and maintaining the seal around dendritic cell filopodia.

These data also provide support for the importance of sequences upstream of the PDZ binding domain in dictating target PDZ domains and subsequent activity. Both the GSIV (Ex7) and ITVV (Ex8) sequences bind all three targets investigated, MAGI-1b, PICK1 and PSD95. However, the presence of the upstream 9 unique amino acids from hCAR^Ex8^ alters the resulting interaction such that PICK1 does not interact, PSD-95 does interact to localize at cellular junctions, and MAGI-1b results in degradation of the hCAR^Ex8^ protein. In contrast, the 22 amino acids from hCAR^Ex7^ allow a stable interaction between these three proteins and the ITVV PDZ-sequence from hCAR^Ex8^ resulting in co-localization at the junctions of cells. The exact mechanism requires further investigation and will lead to a greater understanding of this important class of protein interaction domains.

In summary, human airway epithelia express several isoforms of hCAR. Importantly, human CAR^Ex8^ localizes to the apical surface where it may play a key role in the initiation of adenoviral, and potentially coxsackievirus, pulmonary infection. We propose a model for the regulation of this localization based upon isoform-specific PDZ binding domain interactions with MAGI-1b. Although PDZ based interactions are known to be key regulators of membrane microdomain structure, stability and signaling, we have shown that this interaction may also regulate protein expression. Furthermore, PDZ based interactions are influenced by several factors, including PDZ binding domain and upstream sequence context. Affinity of interaction between the PDZ domain and PDZ binding domain may be higher or lower depending on surrounding sequences resulting in the specificity of interaction. Surprisingly, either interacting partner can be dominant in dictating the result of the interaction (i.e. junctional trafficking versus degradation). Further elucidation of these mechanisms may provide a novel target for either down regulation of the adenovirus receptor to limit viral infection or alternatively up regulation for the purpose of adenoviral-based therapies.

## Materials and Methods

### Ethics statement

This study was conducted according to the principles expressed in the Declaration of Helsinki. The study was approved by the Institutional Review Board of the University of Iowa (IRB ID No. 9507432). Primary human airway epithelia were isolated from discarded and deidentified trachea and bronchi of donor lungs. This study used discarded lung tissue, thus the IRB deemed consent was not needed.

### Materials and constructs

FLAG M2 antibody (Ab) was purchased from Sigma (F3165, St. Louis, MO), mouse anti-CD55 (DAF) was from BD Pharmingen (555691, San Jose, CA), mouse anti-Ezrin was from Santa Cruz (SC-58758, Santa Cruz, CA), mouse anti-acetylated α-tubulin was from Sigma (St. Louis, MO), Alexa-488 and -568 conjugated goat anti-mouse or anti-rabbit Abs, mouse and rabbit anti-GFP were from Molecular Probes (Eugene, OR). RmcB Ab (CRL-2379, ATCC, Manassas, VA) was produced by the University of Iowa Hybridoma Core. Rabbit anti-hCAR 1605 was produced in rabbits immunized with a GST fusion to the intracellular c-terminus (aa 261–365) as previously described [40]. Rabbit anti-hCAR^Ex7^ 5490 and Rabbit anti-hCAR^Ex8^ 5678 were produced in rabbits immunized with peptides of 13 c-terminal amino acids (CVMIPAQSKDGSIV and FKYPYKTDGITVVC respectively). COS-7 cells were from ATCC (Manassas, VA), and maintained under standard culture conditions (D-MEM with 10% FCS, penicillin and streptomycin). CHO-K1 cells were from BD Biosciences (Franklin Lakes, NJ) and maintained under standard culture conditions (D-MEM with 10% FCS, supplemented with tetracycline L-glutamine, penicillin and streptomycin). Ad serotype 5 containing the β-galactosidase (Ad-βGal), eGFP, RFP (peGFP-N1, pDSRed1, Clontech, Palo Alto, CA), or hCAR gene have previously been described [41], [42]. The University of Iowa Gene Transfer Vector Core produced all viruses. Several cDNAs were kind gifts from the following investigators: hCAR was from Ronald Crystal; peGFP-MAGI-1b was from Irina Dobrosotskaya; PSD-95-GFP was from David Bredt. The cDNA for PICK1-GFP has previously been described [43] and MAGI-1b-CMV-myc was subcloned and contained aa 642-1287.

### Web programs

We performed a BLAT search with the mouse sequence (www.genome.ucsc.edu) to determine the human sequence for exon 8. Comparison of hCAR P343 to mCAR A343 using Conseq software identified this amino acid as an exposed or buried residue respectively with a non-structural role. Conservation could not be determined due to insufficient data. The score assigned by Conseq was validated using PolyPhen (http://www.bork.embl-heidelberg.de/polyPhen/).

### Cloning of hCAR^Ex8^ and site directed mutagenesis

hCAR^Ex8^ was cloned from RNA isolated from primary human airway epithelia (Qiagen, Valencia, CA; hCAR^Ex8^F: 5′GCGAATTCGCCACCATGGCGCTCCTGCTCTGCTTCG and hCAR^Ex8^R: 5′GTGGATCCTTATACAACTGTAATTCCATC). The fragment was digested with EcoRI and BamHI and cloned into pcDNA3.1(-) or an Ad5-CMV shuttle plasmid. DNA sequencing and Western blot confirmed the expected protein. hCAR^Ex8^ was modified with two FLAG tags (DYKDDDDK) added between amino acid 22 and 23, similar to previously described modifications in hCAR^Ex7^. Site-directed mutagenesis was performed to generate hCAR^Ex7/8^ according to manufacturer's standard protocol (Stratagene, Cedar Creek, TX) with the following primer: GSIV-ITVV, 5′-CCAGCACAGAGCAAGGATATCACTGTAGTATAGGGATCCGAGCTC.

Site-directed mutagenesis was performed to generate CAR^Ex8/7^ according to manufacturer's standard protocol (Stratagene, Cedar Creek, TX) with the following primer: ITVV-GSIV, 5′-CCTTACAAGACTGATGGAGGTTCAATTGTATAAGGATCAAGGGTG

Site-directed mutagenesis was performed to generate hCAR^Ex8-PDZ^ according to manufacturer's standard protocol (Stratagene, Cedar Creek, TX) with the following primer: ITVV-*TVV, 5′-CCTTACAAGACTGATGGATAAACAGTTGTATAAGGATCAAGGGTGG.

### Human airway epithelia

Primary human airway epithelia were isolated from trachea and bronchi of donor lungs and seeded onto collagen coated, semi-permeable membranes (Millipore, Bedford, MA) and grown at the air-liquid interface as previously described [44], [45]. Approximately two weeks after seeding, cultures were well-differentiated and attained a measurable transepithelial resistance. To augment endogenous hCAR expression, epithelia were transduced with adenovirus carrying hCAR^Ex7^ or hCAR^Ex8^ from the basolateral surface as previously described [23], [41].

### Amaxa transfection

Primary airway epithelial cells seeded on plastic were trypsinized, washed and cells electroporated with 2.5 µg of plasmids encoding hCAR^Ex7^, hCAR^Ex8^ or eGFP using the Amaxa Nucleofector I (Amaxa Inc, Walkersville, MD) according to manufacturer's standard protocol for primary mammalian epithelial cells (VPI-1005, program T-20). Approximately 3×10^5^ cells were seeded onto collagen coated, semi-permeable membranes as described above. Epithelia were infected with Ad-β-gal (MOI 10 pfu/cell) for 1 hr at 37°C, washed twice and lysed 48 hr post infection. β-galactosidase expression per mg protein was determined as previously described [42].

### Adenovirus infection

Chinese hamster ovary cells were plated in 24 well dishes and transfected using Lipofectamine 2000 (Invitrogen, Carlsbad, CA) following the manufacturer's protocol. Twenty-four hours after transfection cells were infected with Ad serotype 5 containing the β-galactosidase gene (Ad-βGal) (MOI 100) for 1 hr at 37°C. 48 hours later cells were lysed and β-galactosidase expression per mg protein was determined as previously described [42].

### Co-transfection in COS-7 cells

COS-7 cells were electroporated by standard methodologies. Briefly, 10 million cells were mixed with 20 µg of plasmid DNA for single transfection, 15 µg of each DNA for double transfections, or 10 µg of each for triple transfections, in 400 µl of cytomix (120mM KCl, 0.15mM CaCl_2_, 10mM K2HPO4, 10mM KH2PO4, 25mM HEPES, 2mM EGTA, 5mM MgCl2, 2mM ATP and glutathione) and put in an electroporation cuvette (Bio-Rad Laboratories, Hercules, CA) for 30 minutes on ice. After electroporation, cells were seeded onto 10cm dishes for immunoprecipitation (IP) and collagen coated glass chamber slides for immunofluorescence studies 2 days later.

### Immunostaining

COS-7 cells grown on collagen coated chamber slides or airway epithelia were washed once with PBS, fixed with 4% paraformaldehyde, permeabilized with 0.1% Triton X-100, and blocked with 2% BSA in SuperBlock (Pierce, Rockford, IL). Cells were incubated with primary Ab, washed extensively and incubated with goat anti-mouse Alexa-568 secondary Ab. After washing, slides were coverslipped with Vectashield mounting media (Vector Laboratories, Inc, Burlingame, CA). Images were acquired with a BioRad MRC-1024 Laser Scanning Confocal Microscope (Hercules, CA) mounted on a Nikon E600 microscope (Melville, NY) using a 60X oil immersion lens. Fluorescence imaging was performed on an Olympus IX71 X-Cite 120 fluorescence microscope (Center Valley, PA) followed by quantitation using Image J.

### Quantitative real time PCR

RNA was isolated from primary airway cultures greater than 2 weeks old or primary lung tissue using TRIzol with the Pure Link RNA kit (Invitrogen). cDNA was synthesized using RT2 EZ First Strand Kit (SA Biosciences). Primers and probes for the seventh or eighth exon of *CXADR* were designed with Primer Express software (Applied Biosystems). The sequences for hCAR^Ex7^ were: hCAR^Ex7^-F, 5′-TGCCAGAAGCTACATCGGCAGTAA; hCAR^Ex7^-R, 5′-ATAGACCCATCCTTGCTCTGTGCT; hCAR^Ex7^ probe, 5′-AAGTCGAATGGGTGCGATTCCTGTGA (5′ FAM, 3′ TAMRA-SP); PCR product 141bp. The sequences for the hCAR^Ex8^ were: hCAR^Ex8^-F, 5′-AGGGAAGATGTGCCACCTCCAAA; hCAR^Ex8^-R, 5′-CAACTGTAATTCCATCAGTCTTGTAAG; hCAR^Ex8^ probe, 5′-ACTGCCAGAAGCTACATCGGCAGTAA (5′ FAM, 3′ TAMRA-SP); 165bp. Samples were run using TaqMan Fast Universal PCR Master Mix (Applied Biosystems) on a 7500 Fast Real-Time PCR System (Applied Biosystems). Transcript number was quantitated by plasmid standard curve. Abundance relative to 18s (Invitrogen, 115HM-02) or hGAPDH (Invitrogen, 100H-02) as the reference gene confirmed the presence of significantly more hCAR^Ex7^ transcripts than hCAR^Ex8^.

### Immunoprecipitation and Western blot

Cells from two 100mm plates were placed on ice, washed once with ice cold PBS, and lysed with lysis buffer (50mM Tris-HCl, pH 7.5, 137mM NaCl, 1% Triton X-100, 5mM EDTA, 1mM EGTA, protease inhibitors (10 µg/ml) leupeptin, aprotinin, pepstatin, and 1mM phenylmethylsulfonyl fluoride) by rocking at 4°C. Cells were scraped, sonicated 5 times and spun in a microcentrifuge at full speed for 10 minutes. For co-immunoprecipitation, supernatant was incubated with the indicated Ab with rotation at 4°C overnight. Protein A or G conjugated sepharose (Amersham Biosciences, Uppsla Sweden) was added for 1-2 hours followed by a wash with lysis buffer, 10% lysis buffer in TBS (50mM Tris-HCl, pH 7.5, 137mM NaCl), and TBS. Beads were suspended in loading buffer (4% sodium dodecyl sulfate, 100mM dithiothreitol, 20% glycerol, 65mM Tris, pH 6.8, 0.005% bromophenol blue) and proteins were separated by SDS-poly acrylamide gel electrophoresis. Gels were transferred to a polyvinylidene difluoride membrane (Millipore, Bedford, MA), blocked with 5% BSA, washed, probed with primary Ab as indicated, followed by washing and incubation with protein A or G conjugated HRP (Pierce, Rockford, IL). Bands were detected with ECL reagents (Pierce, Rockford, IL) and imaged on the EpiChemi^3^ Darkroom (UVP Inc, Upland, CA).

## Supporting Information

Figure S1 Endogenously expressed CAREx8 (A, D, G) in polarized human airway epithelia localizes above ZO-1 (B, C) and co-localizes with the apical protein ezrin (E, F, G). Sections are shown in X-Y (A-F) or X-Z (G) axes. Confocal microscopy (60x oil immersion).
(9.08 MB TIF)

Figure S2 Quantitative RT-PCR primers for CAREx7 or CAREx8 are specific. Primary airway were either mock transduced or transduced with adenovirus carrying the gene for CAREx7 or CAREx8. RNA was isolated 36 hours later and subjected to isoform specific quantitative RT-PCR. Under mock conditions there was more endogenous CAREx7 than CAREx8. Epithelia transduced with CAREx7 or CAREx8 showed increased trascript levels but did not increase transcript levels of the other isoform.
(2.91 MB TIF)

Figure S3 Expression of exogenous CAREx8 in polarized human airway epithelia mediates five-fold greater Ad-β-Gal gene transfer than endogenous expression (mock transduced cells followed by Ad-β-Gal). *p<0.0001 Ad-CAREx8 vs. Mock/Ad-β-Gal or Mock/no virus. p = 0.03 Mock/Ad-β-Gal vs. Mock/no virus.
(2.50 MB TIF)
