# Supplementary figures and images for: Isoform-Specific Regulation and Localization of the Coxsackie and Adenovirus Receptor in Human Airway Epithelia
Source: PLoS One. 2010 Mar 26;5(3):e9909. doi: 10.1371/journal.pone.0009909 (PMC2845650; doi:10.1371/journal.pone.0009909)

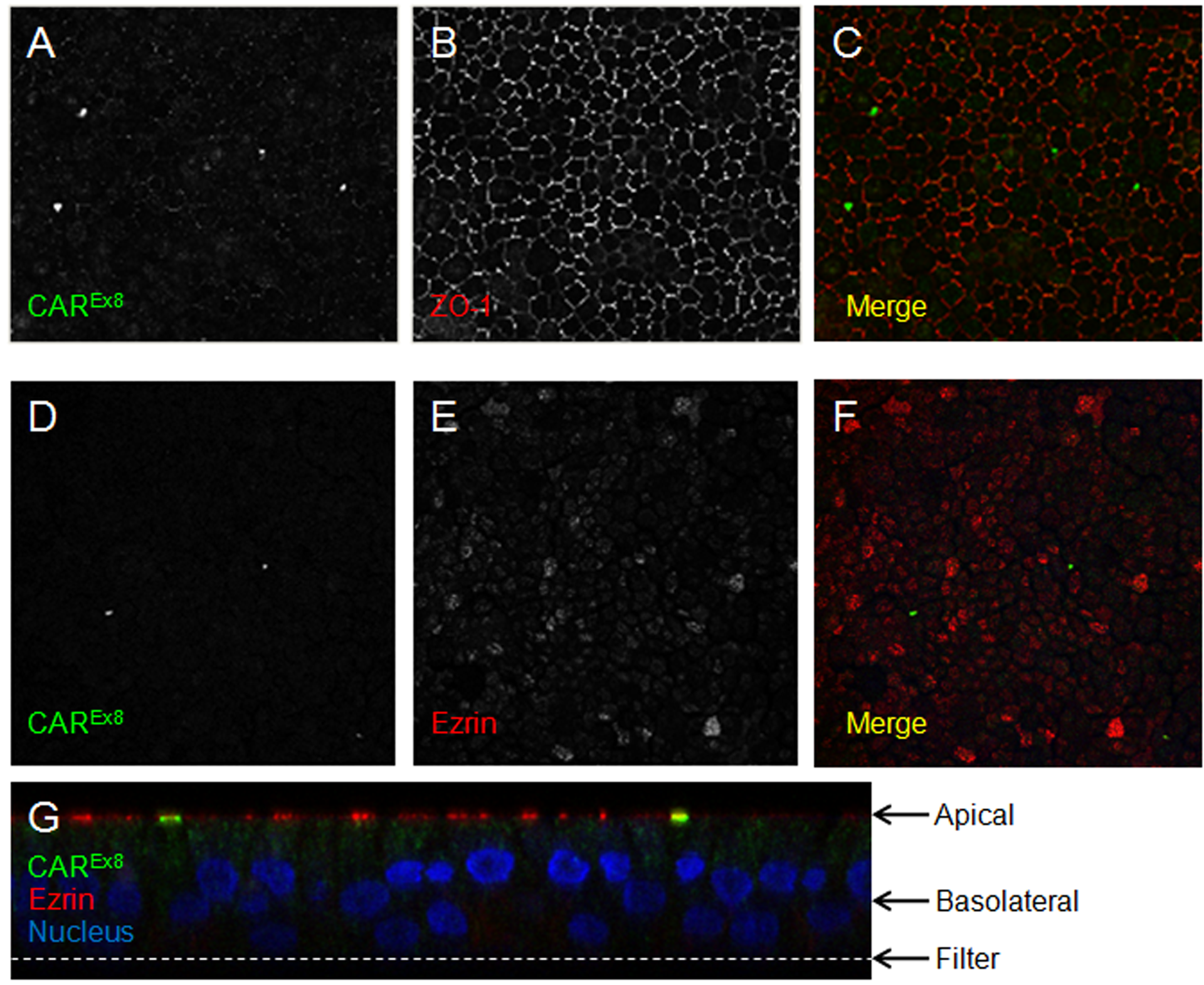

Supplement: Figure S1 — Endogenously expressed CAREx8 (A, D, G) in polarized human airway epithelia localizes above ZO-1 (B, C) and co-localizes with the apical protein ezrin (E, F, G). Sections are shown in X-Y (A-F) or X-Z (G) axes. Confocal microscopy (60x oil immersion). (9.08 MB TIF) [file pone.0009909.s001.tif]

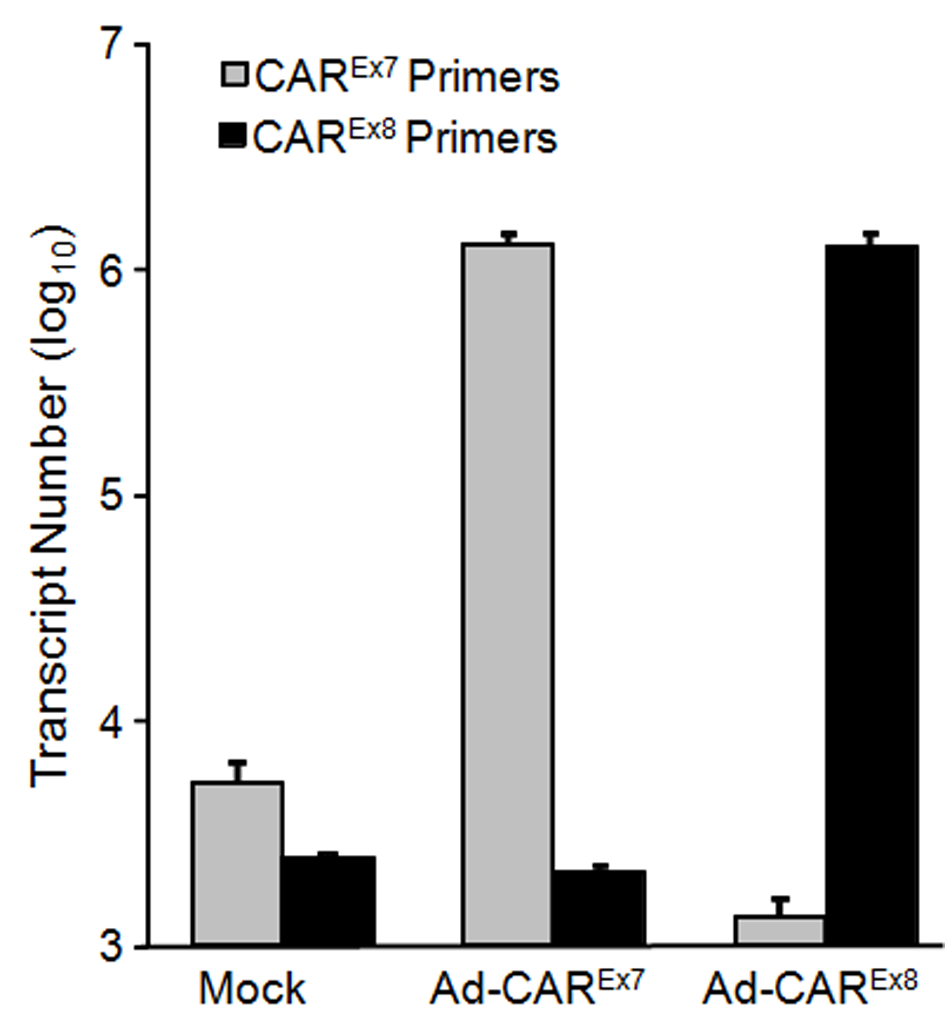

Supplement: Figure S2 — Quantitative RT-PCR primers for CAREx7 or CAREx8 are specific. Primary airway were either mock transduced or transduced with adenovirus carrying the gene for CAREx7 or CAREx8. RNA was isolated 36 hours later and subjected to isoform specific quantitative RT-PCR. Under mock conditions there was more endogenous CAREx7 than CAREx8. Epithelia transduced with CAREx7 or CAREx8 showed increased trascript levels but did not increase transcript levels of the other isoform. (2.91 MB TIF) [file pone.0009909.s002.tif]

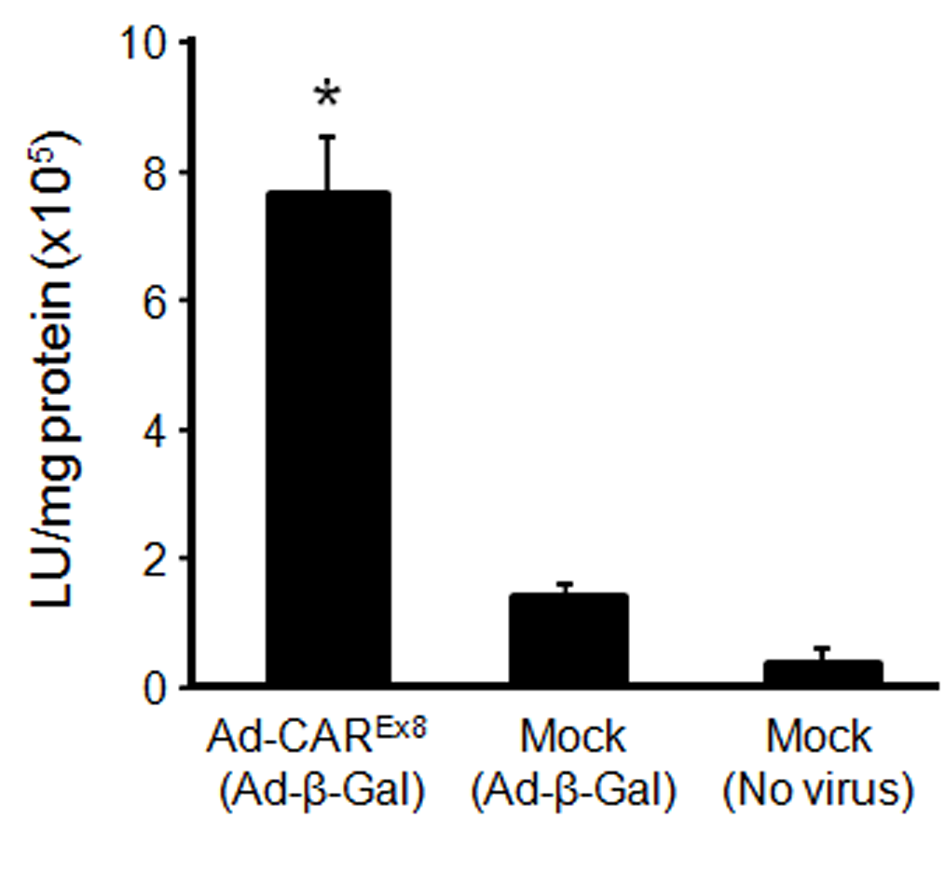

Supplement: Figure S3 — Expression of exogenous CAREx8 in polarized human airway epithelia mediates five-fold greater Ad-β-Gal gene transfer than endogenous expression (mock transduced cells followed by Ad-β-Gal). *p<0.0001 Ad-CAREx8 vs. Mock/Ad-β-Gal or Mock/no virus. p = 0.03 Mock/Ad-β-Gal vs. Mock/no virus. (2.50 MB TIF) [file pone.0009909.s003.tif]
